# Collaborative care treatment for major depressive disorder

**DOI:** 10.3389/fpsyt.2026.1798572

**Published:** 2026-07-01

**Authors:** Chase Walker, Virna Little, Jennifer Lyons

**Affiliations:** 1 JG Research and Evaluation, Bozeman, MT, United States; 2 Concert Health, San Diego, CA, United States; 3 Advent Health, Altamonte Springs, FL, United States

**Keywords:** collaborative care, collaborative care delivery, depression, major depressive disorder, mental health

## Abstract

**Introduction:**

Major Depressive Disorder (MDD) is a burdensome behavioral health condition that is costly and difficult to treat, particularly for patients with severe cases. The Collaborative Care Model (CoCM) has been shown to be effective for moderate depression treatment but less is known about its effectiveness for severe depression. This study analyzes the impact of CoCM treatment on outcomes for depression patients across all ranges of MDD severity at Concert Health.

**Materials and methods:**

Analysis was completed utilizing all closed patient treatment episodes (N = 30,162) at Concert Health between 2018 and 2025. Of these patients, 5,693 began treatment with severe depression. We compare effect sizes for change between baseline and final screener scores across severity levels. Additionally, we utilize logistic regression to complete analyses to understand treatment factors and patient characteristics that are associated with treatment response and remission, as measured by changes in PHQ-9 scores.

**Results:**

The primary analysis showed that patients with severe depression (PHQ-9 > 20) had slightly lower odds of achieving response compared to patients with moderate depression (OR: 0.93). The treatment factors of insurance type, suicide risk, anxiety presence, and touchpoints also had significant effects on the odds of achieving response and remission.

**Discussion:**

The results suggest that CoCM may be effective for patients with severe depression in achieving treatment response. Patients on Medicaid or with more complex conditions such as anxiety presence or elevated risk for suicide may need higher levels of engagement from the care team to achieve response and remission.

## Introduction

Depression is a highly costly and burdensome behavioral health condition that is associated with lower quality of life, lower productivity, and high healthcare costs (1, 2). One recent study estimated that approximately 19.8 million adults in the US had major depressive disorder (MDD), with an incremental societal burden of MDD of $333.7 billion, while another study found that an increase in depression severity was associated with an average increase in total per member per month healthcare spending of $608 (3, 4). Given the high prevalence of major depression and it’s negative impacts on society, it is important to understand and prioritize interventions and care models that offer individuals with MDD valid treatment options. In this paper we evaluate outcomes for patients with severe depression receiving treatment through the Collaborative Care Model (CoCM) from the national telehealth CoCM provider, Concert Health.

CoCM is an evidence based model to identify and treat patients with behavioral health conditions in healthcare settings and is supported by many randomized controlled trials as well as an additional body of growing research (5, 6). CoCM specifically integrates behavioral health clinicians and psychiatric consultants into primary healthcare settings, thus creating a team that works to treat behavioral health conditions in patients. There have been a number of meta-analyses that have demonstrated the effectiveness of CoCM in managing depression through improvements in treatment adherence, symptom reductions, and patient satisfaction (7–9). Additionally, the model has been shown to be highly effective for managing anxiety disorders and can be extended across a diverse set of populations and care delivery settings (10–12).

Although the evidence base is strong, in particular compared to care as usual, CoCM has for many years been considered a model for treating individuals primarily with “mild to moderate” symptoms. Less is understood about patients with severe depression or more complex clinical presentations, who were sometimes excluded from CoCM historically or considered to need more intensive treatment. This is supported by prior research, which has found that patients with higher levels of depression severity had worse clinical outcomes despite higher levels of treatment, leading to the conclusion that new classifications of depression be used based on clinical severity, course of illness, and treatment experience (13). Additionally, other patient characteristics and the presence of elevated anxiety are associated with worse outcomes (13, 14). Despite this, there is evidence that for individuals who eventually achieve clinical remission that rapid response is most pronounced in severely depressed patients in CoCM (15). While this study included an idealized patient population, in that it included only patients who had achieved clinical remission, it demonstrates that under the right setting that severely depressed patients may improve.

More recently, those practicing in CoCM have started to find that best practice was not to exclude individuals based on score and, in fact, that those individuals with elevated scores often saw significant symptom reduction in CoCM and greater than those in traditional care (16). Simultaneously a growing body of evidence has supported CoCM to treat individuals at risk for suicide (17, 18), Post Traumatic Stress Disorder and Bipolar disorder (19–21). Despite this growing body of evidence, there is little research that we are aware of which specifically examines and compares outcomes in CoCM for patients with severe symptoms compared to a typical patient with moderate depression, particularly within a real world patient population. Given the field experience and expanding research support, this article supports the ability to not only treat but significantly improve the symptoms of those with what would be considered severe symptoms of depression.

## Materials and methods

This study is a retrospective observational analysis of adults receiving outpatient treatment for depression through CoCM at Concert Health. Concert Health is a national behavioral health medical group that provides primarily telehealth delivered CoCM to primary care, pediatric, and women’s health providers and organizations across 21 states. A patient is referred to CoCM by their primary care provider based on standardized screening and symptoms for behavioral health conditions. CoCM is patient centered, providing multiple treatment choices for patients along with being measurement-based care, seeking to reduce symptoms by 50% in the first few months of treatment. CoCM is a monthly case rate, versus individual fee for service visits, and was recognized in 2017 by CMS with dedicated CPT codes. These codes are now covered by commercial plans as well as on the Medicaid fee schedules in 34 states.

Data were collected from Concert Health’s electronic patient registry, which maintains records of all open and completed patient episodes across all states and medical system partners. Patients are entered into the system when they are referred to Concert Health by their healthcare provider and begin receiving treatment with their CoCM team. Data includes patient records specific only to collaborative care. This means that medical records specific to their healthcare provider, such as other health diagnoses and detailed demographic information, are stored in the partner medical system’s EMR and not accessible for this review to Concert. The online patient registry includes basic demographic characteristics, information on patient touchpoints through treatment, and treatment measurement factors such as PHQ-9 and GAD-7 scores, as well as suicide risk screening scores and classifications. The utilization of the patient registry allows for clinicians to adhere to measurement based care, which is a key mechanism of CoCM and allows for the continual tracking and assessment of patient outcomes by the CoCM team.

**Figure 1:**
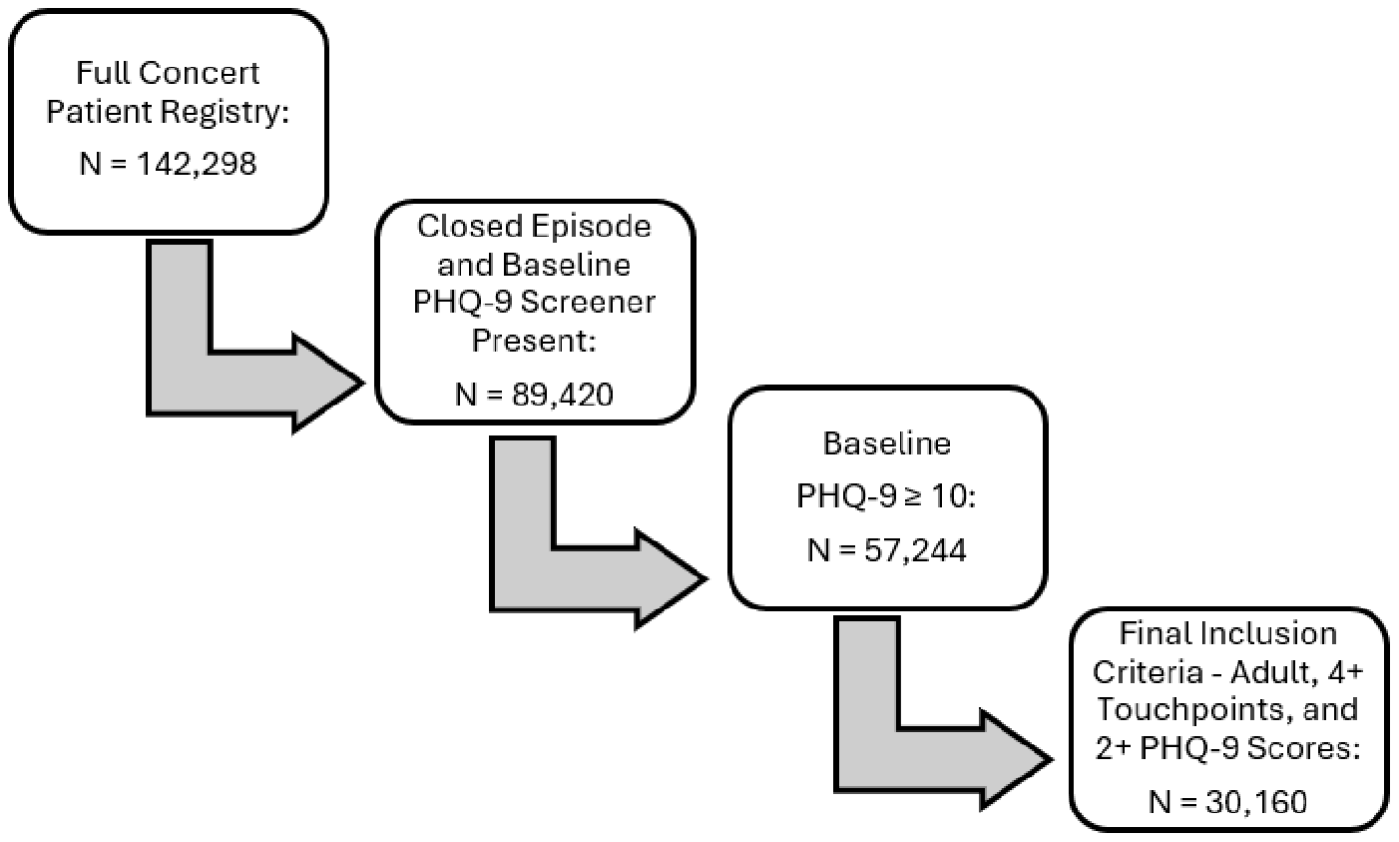
Patient inclusion criteria.

The sample included all completed Concert Patient episodes from December 2018 to November 2025. Inclusion criteria required a patient to have completed a baseline PHQ-9 screening with a PHQ-9 score of greater than or equal to 10 (considered the threshold for clinically significant depression), as well as all relevant treatment measurement factors having complete data. A patient episode is defined as a patient having an enrollment start date and a defined enrollment end date, with all episodes meeting these criteria included regardless of discharge reason. If the patient does not have a defined enrollment end date, their episode is still open, and they were removed from the sample as these patients are still receiving treatment. After initial screening, final inclusion criteria required patients to have completed at least four clinical touchpoints and completed at least two PHQ-9 screenings to ensure analysis of patients who received treatment and calculation of outcome variables. The final sample includes 30,160 patients, 5,693 of which had severe baseline depression and 24,467 of which had moderate or moderately severe depression. The sample only includes adult patients, therefore any patients with an age at enrollment less than 18 were excluded. A flow chart demonstrating our inclusion criteria is displayed in **Figure 1**.

The standard definitions of improvement in the Healthcare Effectiveness and Data Information Set (HEDIS) implemented by the National Committee for Quality Assurance (NCQA) are that response is a 50% or greater reduction in score and remission is defined as a follow up score of < 5 on the PHQ-9 (22). Therefore, we utilize depression response (50% or greater PHQ-9 reduction) and remission (PHQ-9 < 5) from baseline to final PHQ-9 score prior to discharge as outcomes in statistical models and calculate effect sizes of PHQ-9 change between baseline and final score before discharge. Outcome variables are generated as binary variables, where patients who achieve response or remission classified as 1, and those that did not achieve response or remission classified as a 0.

We compare effect sizes for change in PHQ-9 scores from baseline to final within each severity category using Cohen’s D, and test for statistical significance with paired t-tests. Statistics are broken out by depression severity level. Multivariate logistic regression models are used to test the associations between the two outcome measures and clinical variables and patient characteristics. Separate models are used for response and remission, with the same set of covariates being utilized in each model. Covariates include age group, insurance type, average number of clinical touchpoints per week per patient, anxiety presence, and suicide risk flag.

Age group is a prespecified variable present in the Concert patient registry and includes the age categories of 18-30, 31-45, 45-64, and 65 +. Insurance types include Medicaid, Commercial, Medicare, and Medicare Advantage. The number of clinical touchpoints per week was generated utilizing enrollment length and clinical touchpoint data from the patient registry. A clinical touchpoint is any contact with a clinician greater than or equal to five minutes. This variable was generated to account for substantial variation in timing and spread of clinical touchpoints across episode lengths for patients. While the goal of CoCM is frequent engagement and early improvement within the first 3 to 4 months of engagement, many patients experience longer or shorter treatment times and engage at frequencies that may vary by provider, medical system, and patient needs or preferences. Some patients may receive a similar “dose” (with the dose being clinical touchpoints) of treatment but have substantial differences in total episode length that leads to the effect of the dose being minimized by a long episode length and infrequent clinical engagement. To account for this variation, we calculate touchpoints per week as a measure of “dose”, where patients with more frequent engagement during their episode will have a higher number of touchpoints per week and patients with less frequent engagement will have a lower number of touchpoints per week.

We also include several variables that serve as indicators of patients who may require higher levels of treatment due to additional case complexity of presentation of other mental health symptoms. Anxiety presence is a variable based on the clinical presentation of anxiety, as measured by screener scores of the GAD-7 for anxiety. All patients, regardless of primary diagnosis, are administered both a PHQ-9 and GAD-7 at baseline and often times both frequently throughout treatment. Any patient in our population who screened at a clinical presentation of anxiety on the GAD-7 (> 10) are categorized as having clinically relevant anxiety, which is used essentially as a proxy for patients with potentially comorbid conditions, who may be more difficult to treat. Finally, suicide risk flag is also tracked within the Concert patient registry, and tracks patients current suicide risk level based on screening information from the PHQ-9, C-SSRS, and clinician assessments. Categories include No Risk, Historical Risk, At Risk, and High Risk. Historical risk indicates patients who had previously been flagged positive for suicide but had symptomatic and clinically meaningful improvement and subsequent loss of suicide risk. We include a binary variable for suicide risk that represents whether a patient had any suicide risk at any point in treatment. While this variable may minimally bias the model, as suicide risk is not always flagged at baseline, we believe that inclusion is relevant, as suicidality is common amongst patients with depression and will lead to a specialized treatment pathway and potentially impact outcomes.

Results from logistic regressions are presented as odds ratios. All analysis was completed using R and RStudio (23). A research protocol was submitted to Western IRB and determined to be exempt under 45 CFR § 46.104(d) (4).

## Results

### Demographics

**Table 1** summarizes the patient population, broken out by baseline depression severity levels. The total analysis sample includes 30,160 closed patient episodes. The first two rows display the outcome variables, response and remission, while the following rows display all covariates included in statistical models. Categorical variables display n values and percentages, while continuous variables are shown with median and interquartile values.

**Table 1:** Descriptive statistics.

Characteristic	Moderate (10-14) N = 13,095*^1^*	Moderately Severe (15-19) N = 11,372*^1^*	Severe (20-27) N = 5,693*^1^*	p-value*^2^*
PHQ-9 Response	7,413 (57%)	6,273 (55%)	2,940 (52%)	<0.001
PHQ-9 Remission	5,552 (42%)	3,388 (30%)	1,172 (21%)	<0.001
Age Group				<0.001
18–30 years	3,346 (26%)	3,154 (28%)	1,508 (26%)	
31–45 years	3,833 (29%)	3,262 (29%)	1,769 (31%)	
46–64 years	3,419 (26%)	3,304 (29%)	1,795 (32%)	
65+ years	2,497 (19%)	1,652 (15%)	621 (11%)	
Anxiety Presence				<0.001
Yes	8,195 (63%)	7,977 (70%)	4,141 (73%)	
No	4,900 (37%)	3,395 (30%)	1,552 (27%)	
Insurance				<0.001
Commercial	7,346 (56%)	6,189 (54%)	2,884 (51%)	
Medicaid	2,838 (22%)	2,968 (26%)	1,728 (30%)	
Medicare	1,178 (9.0%)	839 (7.4%)	391 (6.9%)	
Medicare Advantage	1,733 (13%)	1,376 (12%)	690 (12%)	
Suicide Risk Flag				<0.001
Yes	2,909 (22%)	3,990 (35%)	2,991 (53%)	
No	10,186 (78%)	7,382 (65%)	2,702 (47%)	
Touchpoints per Week	0.45 (0.35, 0.56)	0.46 (0.36, 0.57)	0.48 (0.37, 0.61)	<0.001

*^1^*n (%); Median (Q1, Q3).

*^2^*Pearson’s Chi-squared test; Kruskal-Wallis rank sum test.

Across the three severity levels, PHQ-9 Response rates are relatively similar, with slight decline as depression severity increases. Remission rates decline substantially as severity increases, with 42% of Moderate depression patients achieving remission while only 21% of Severe depression patients achieve remission. Similarly, the prevalence of anxiety and suicide risk increase with higher baseline depression severity levels.

### Symptom change

**Figure 2:**
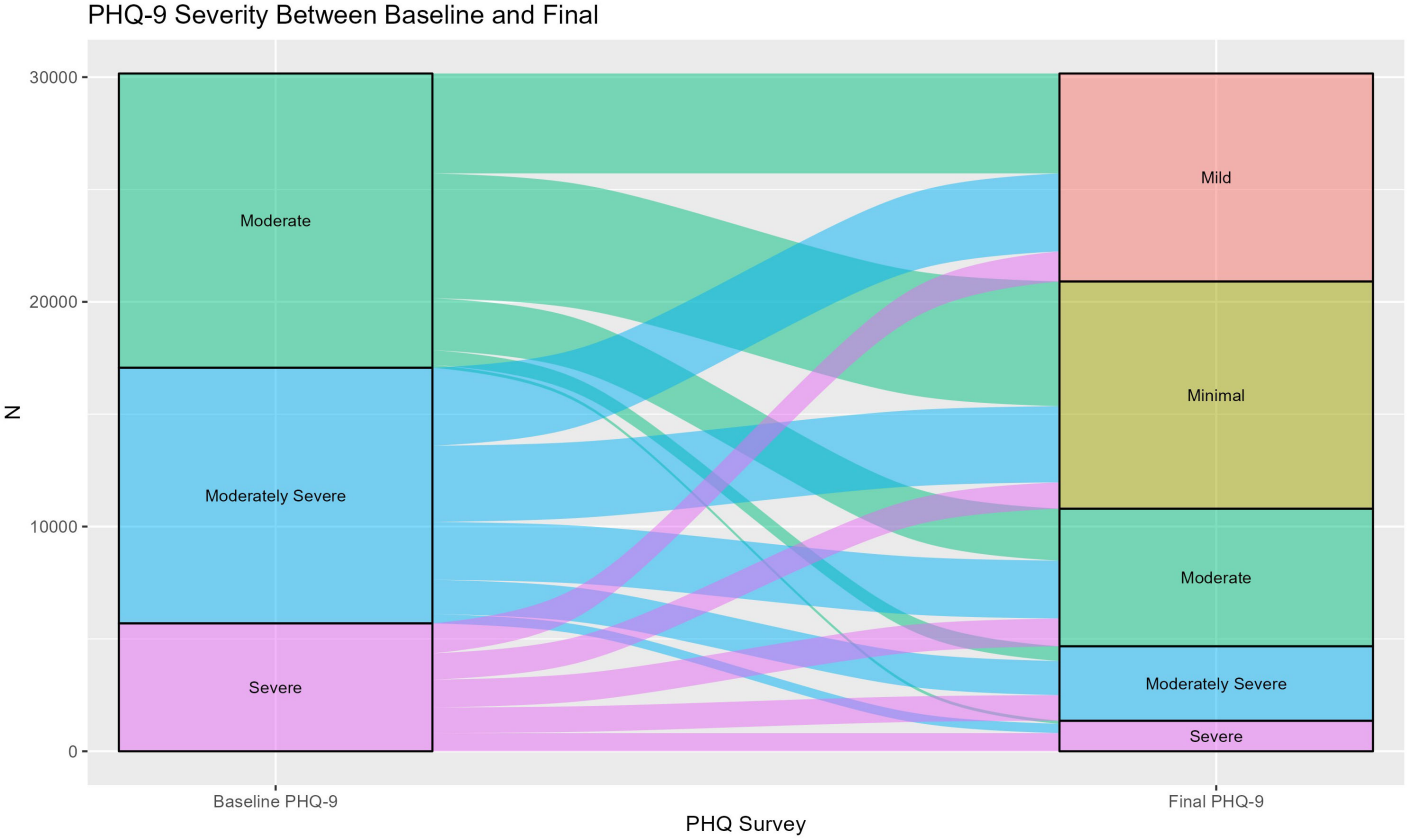
Patient alluvial chart - baseline to final.

**Figure 2** is an alluvial diagram that shows the flow of patients between depression severity categories from baseline to final score. In general, more patients improve over treatment, with a smaller subset of patients remaining at the same severity level, and an even smaller subset of patients seeing declining outcomes. At final PHQ-9 prior to discharge, over 60% of the patient population had improved to mild or minimal depressive symptoms.

**Table 2** shows average baseline and final PHQ-9 scores across baseline depression severity classifications. On average, patients see roughly a 50% reduction between baseline and final across severity classes, with the change being statistically significant within each group. Additionally, effect sizes are large in magnitude within each group (Cohen’s D; Severe: 2.12, Moderately Severe: 2.00, Moderate: 1.65).

**Table 2:** T-tests and effect size - baseline to final PHQ-9 comparison by severity.

T-tests and effect size - baseline to final PHQ-9							
Baseline severity class	Avg. PHQ-9 baseline	Avg. PHQ-9 final	*t*	*df*	*p*	*d*	95% CI
Severe	21.85	11.23	118.01	5,692	<.001***	2.12	[2.07, 2.17]
Moderately Severe	16.84	8.5	155.42	11,371	<.001***	2.00	[1.97, 2.04]
Moderate	12.03	6.3	139.05	13,094	<.001***	1.65	[1.62, 1.68]

Effect size and 95% CI is Cohen’s D.

### Treatment response comparisons

**Table 3** displays results, as odds ratios, from the logistic regression models for response and remission. The primary covariate of interest is baseline PHQ-9 classification, which measures relative odds of response or remission across moderate, moderately severe, and severe baseline depression levels. In the response model, there are no statistically significant differences in the odds of achieving response for moderately severe patients while the odds for severe patients are slightly lower relative to patients with baseline moderate depression., The magnitude of difference in odds of achieving response relative to the reference category of Moderate baseline depression is minimal as well (Moderately Severe OR: 1.00 (0.95, 1.06), Severe OR: 0.93 (0.87, 0.99)). In other words, all else being equal, patients have roughly similar odds of achieving clinically meaningful depression response, with slightly lower for patients with severe depression.

**Table 3:** Model results – odds ratios for response and remission outcomes.

Characteristic	Response model (50% improvement)			Remission model (PHQ-9 < 5)		
	OR	95% CI	p-value	OR	95% CI	p-value
Baseline PHQ-9 Class						
Moderate (10-14)	—	—		—	—	
Moderately Severe (15-19)	1.00	0.95, 1.06	0.9	0.61	0.58, 0.65	**<0.001**
Severe (20-27)	0.93	0.87, 0.99	**0.027**	0.40	0.37, 0.43	**<0.001**
Anxiety Presence						
Yes	—	—		—	—	
No	1.13	1.07, 1.18	**<0.001**	1.19	1.13, 1.25	**<0.001**
Age Group						
18–30 years	—	—		—	—	
31–45 years	1.01	0.95, 1.07	0.8	1.05	0.98, 1.12	0.15
46–64 years	1.02	0.95, 1.08	0.6	1.04	0.97, 1.11	0.3
65+ years	1.71	1.54, 1.90	**<0.001**	1.59	1.42, 1.78	**<0.001**
Insurance						
Commercial	—	—		—	—	
Medicaid	0.74	0.70, 0.78	**<0.001**	0.70	0.66, 0.74	**<0.001**
Medicare	0.71	0.64, 0.80	**<0.001**	0.81	0.72, 0.91	**<0.001**
Medicare Advantage	0.72	0.66, 0.79	**<0.001**	0.75	0.68, 0.83	**<0.001**
Touchpoints per Week	1.59	1.40, 1.81	**<0.001**	1.65	1.44, 1.89	**<0.001**
Suicide Risk						
No	—	—		—	—	
Yes	0.77	0.73, 0.81	**<0.001**	0.77	0.73, 0.82	**<0.001**

CI, Confidence Interval; OR, Odds Ratio. Reference categories are displayed in the first row for categorical variables.

Bold values indicate statistical significance.

In the remission model, the odds of achieving remission are statistically significantly lower for Moderately Severe and Severe depression at baseline, relative to Moderate (Moderately Severe OR: 0.61 (0.58, 0.65), Severe OR: 0.40 (0.37, 0.43)). This finding is expected, as remission rates are lower as baseline depression is higher.

Relative to the reference age group of 18-30, all middle adult age groups in both models have no significantly different odds of improvement, while adults over age 65 have significantly higher odds of improvement in comparison (Response OR: 1.71 (1.54, 1.90), Remission OR: 1.59 (1.42, 1.78). Patient insurance also plays a significant role in treatment improvement, with Medicaid, Medicare, and Medicare Advantage patients having lower odds of improving relative to patients who are commercially insured.

In addition to the patient characteristics, measures of patient case complexity also play significant roles in the odds of achieving treatment response or remission in care. Patients who begin treatment with anxiety presence, and patients who are at risk of suicide have lower odds of improvement relative to patients who do not present with these additional conditions. Unsurprisingly, patients who are flagged to have a risk for suicide have lower odds of improving relative to patients not flagged (Response OR: 0.77 (0.73, 0.81), Remission OR: 0.77 (0.73, 0.82)).

## Discussion

Our analysis of outcomes for patients beginning CoCM with clinically significant depression symptoms provide evidence that CoCM may be an effective intervention in achieving response to treatment for patients with not only moderate symptoms of depression, but also more severe cases, as rates of response are similar across severity classifications. We also found there to be large effect sizes in measurements of change between baseline and final PHQ-9 across all baseline severity levels. Remission is a more difficult outcome for patients at higher levels of severity to achieve, as we found that the odds of remission decrease significantly as baseline severity is higher, which is an expected outcome. Rates of remission within this population, however, are comparable to rates found in randomized control trials for CoCM, with ranges from 21% to 42%. RCT’s, such as STAR*D and PROSPECT have reported depression remission rates at varying treatment lengths between 13% and 36.8% (STAR*D) and 26.6% to 45.4% (PROSPECT) (24, 25). We find that other treatment and patient level factors were also found to be significantly associated with the likelihood of response and remission, including the presence of baseline anxiety symptomse, patient insurance type, age group, and suicide risk.

Primarily, we provide evidence that treatment outcomes in CoCM are similar across all patients as measured by response (50% or greater reduction in PHQ-9), regardless of whether a patient has severe depression at baseline. While previous literature has shown that patients with higher baseline severity improve somewhat but typically have lower odds of achieving remission through CoCM or need longer treatment doses to achieve clinically meaningful improvement (13, 15, 26), we argue that response is an equally meaningful way to measure the effectiveness of treatment for patients with severe depression despite some disagreement on whether HEDIS measures are optimal. One previous study broadly examined depression improvement across 33 different organizations with variation in depression treatment success criteria, as measured by the PHQ-9 (11). Depression success metrics across these organizations generally look at treatment success as either “response” or “remission”. Overall, the prior literature tends to universally define “remission” as achieving a score of less than five, while it is more divided on the definition of “response” (27–30). For example, “response” has previously been defined as a decrease of 50% or more AND score of <10, a decrease of 5 or more points, and simply just a decrease of 50% or more (11). Carlo et al, found that despite this variation in metrics used, the specific metric is less important than consistency in which metric to use when comparing improvement rates within organizations, as improvement rates vary substantially depending on the metric used.

This challenge was further examined by Coley et al. (31) in Measurement Based Care (MBC) for depression, where the relationship between four different treatment success measures (Response (> 50% reduction), remission (PHQ-9 < 5), effect size (≥ 0.8), and severity adjusted effect size (SAES; ≥ 0.8)) and baseline symptom severity were compared to determine the optimal success measure. They determined that utilizing “response” as a treatment success measure is preferable because it does not favor the magnitude of baseline symptom severity, it indicates improvement that is clinically meaningful, and it is transparent and easy to calculate (22). Additionally, many patients will have treatment resistant depression, particularly if they have high initial symptom severity, making response a more realistic success measure (31).

Defining the correct outcome measure was an important component for this paper focused on severe depression outcomes, and we argue that response measured between baseline and final screening may be preferable because it does not favor the magnitude of baseline symptom severity and is easily translatable and measurable by clinicians in typical care settings, while also demonstrating that treatment is leading to clinically measurable improvement. Regardless of outcome, past research has shown that higher depression severity is associated with longer treatment times, more difficulty achieving remission, frequent symptom relapse, and higher treatment complexity associated with comorbid or chronic health conditions (13, 32, 33).

Consistently with the research just referenced, each of the covariates in both models have significant impacts on the odds of patients achieving response or remission in their treatment episode, with statistical significance and magnitude being fairly consistent, suggesting that regardless of the treatment outcome, these patient factors may be highly important. Of highest clinical relevance is that patients with baseline clinically relevant anxiety symptoms and patients at elevated risk of suicide had significantly lower odds of both response and remission. This is consistent with other literature (14, 26), which has found that increased initial anxiety and bipolar disorder symptoms predicted not achieving remission at six months for severe depression patients in CoCM. Because our suicide variable is simply an indicator of any suicide risk in an episode, results may be susceptible to some bias since it is not always representative of baseline risk. A limitation of the data for this research is the lack of baseline suicide information and inconsistency in reporting. Suicide metrics may be monitored in a variety of ways that can vary from patient to patient and across clinicians, as suicide risk may be flagged by a positive response to question nine of the PHQ-9, through a C-SSRS screening, or by clinician judgement or psychiatric consultation, however these were not consistently tracked or available in the data. We argue that accurate and easily trackable suicide monitoring should be further integrated into clinician practices and EHR systems, as accurate symptom monitoring may improve patient treatment outcomes, streamline care coordination between providers, and allow for more precision in future research.

Patient insurance type is also a significant variable, with severe depression patients who are commercially insured having higher odds of remission relative to patients on Medicaid and Medicare. Similarly, other recent research on CoCM has also found that commercially insured patients are more likely to improve relative to those on Medicaid (34, 35). These findings suggest that clinicians may need to provide more specialized care to higher complexity patients and patients on Medicaid or Medicare, as patients on Medicaid are also more likely to have additional mental health conditions and be at elevated risk for suicide within our sample. Additionally, we also recommend that states explore expanding Medicaid programs to include CoCM billing codes, as not all states include these codes within their Medicaid programs, and states that do have the codes available are able to provide more access to CoCM patients across all insurance types (36).

Finally, our measure of treatment “dose”, the number of clinical touchpoints per week of a care episode, significantly increases the odds of achieving both response and remission, suggesting that patients who engage more frequently with their care team throughout their episode could experience better outcomes, on average. Similarly, in the relatively limited literature on dose response for MDD, there is evidence that there is strong improvement early in treatment, with improvement continuing at a slower pace further into treatment (37). Treatment engagement in CoCM is largely driven by patients and their individual needs or circumstances, and these findings may be influenced by patients who are more motivated to improve and consequently engage in and adhere to treatment, who will likely naturally experience better outcomes as a result. Regardless, clinicians and health systems should continue to consider ways to improve consistent treatment engagement and identify and target patients who have been shown to be more at risk for treatment disengagement (38).

### Limitations

There are several limitations that must be noted. First, this research is from a real-world observation of clinical data, and therefore causal effects of treatment were not possible, meaning any significant effects represent associations and must be interpreted cautiously. Specifically, Any significant effects demonstrate comparisons within the Concert Health patient population between groups and do not contain comparisons to untreated control groups. Additionally, as the Concert Health patient registry has not historically tracked comprehensive patient demographics there is a lack of demographic data (such as race, ethnicity, gender/sex, and socioeconomic status), which would have greatly strengthened our analysis by controlling for potentially confounding factors, evaluating sub-outcomes, and providing more opportunity to potentially inform best practices and population health approaches for diverse sub-populations. We also acknowledge potential bias in our model due to the inclusion of our suicide variable, which may contain some information not collected at baseline. Future research should strive to include detailed demographic information and improve monitoring and data collection for complexity variables such as suicide risk to advance clinical understanding of depression treatment for minorities, underserved populations, and patients with complex cases. Despite these limitations, this research has a major strength of utilizing a large analytical sample and evaluating the effects of CoCM from a single provider integrated across numerous health systems and geographies nationally.

## Conclusions

As the country continues to experience significant challenges with behavioral healthcare access and treatment, in particular with decreased access to evidence based care, understanding ways to increase access and understand ways that difficult to treat populations can improve is important. CoCM is evidence-based treatment that can, and often does, provide same day access. This research expands upon the existing evidence base by highlighting patients with severe depression in a large clinical population of over 30,000 patients, over 5,000 of which had severe baseline depression. We find that in this population, 52% of patients with severe depression achieved treatment response at discharge, which is a similar rate to patients with more moderate depression symptoms. This research demonstrates evidence of the ability of CoCM to effectively reduce symptoms in the “severe” category, as defined by the PHQ-9 score over 20. These findings may further support the continued advancement, support and adoption of CoCM by states, payers, healthcare organizations and providers, as well as provide motivation to continue researching populations with severe depression and complex symptoms further to better understand how treatment can best lead to positive outcomes. Inclusion of patients with PHQ-9 scores over 20 will dramatically increase access to evidence-based treatment for those who need it most.

## Data Availability

The data analyzed in this study is subject to the following licenses/restrictions: Data was accessed from Concert Health’s patient registry and is unavailable publicly. Any questions or requests regarding the data utilized for this manuscript may be directed to the corresponding author.
