# Correction: Collaborative care treatment for major depressive disorder

**DOI:** 10.3389/fpsyt.2026.1925587

**Published:** 2026-07-15

**Authors:** Chase Walker, Virna Little, Jennifer Lyons

**Affiliations:** 1 JG Research and Evaluation, Bozeman, MT, United States; 2 Concert Health, San Diego, CA, United States; 3 Advent Health, Altamonte Springs, FL, United States

**Keywords:** collaborative care, collaborative care delivery, depression, major depressive disorder, mental health

There was a mistake in **Table 2** as published. In the row for Baseline severity class of “Severe”, *d* should be “2.12” instead of “0.83” and the 95% CI should be “2.07, 2.17” instead of “0.80,0.87”. The corrected **Table 2** appears below.

**Table 2:** T-tests and effect size - baseline to final PHQ-9 comparison by severity.

T-tests and effect size - baseline to final PHQ-9							
Baseline severity class	Avg. PHQ-9 baseline	Avg. PHQ-9 final	*t*	*df*	*p*	*d*	95% CI
Severe	21.85	11.23	118.01	5,692	<.001***	2.12	[2.07, 2.17]
Moderately Severe	16.84	8.5	155.42	11,371	<.001***	2.00	[1.97, 2.04]
Moderate	12.03	6.3	139.05	13,094	<.001***	1.65	[1.62, 1.68]

Effect size and 95% CI is Cohen’s D.

The original version of this article has been updated.

